# Effects of CFTR Modulators on *Pseudomonas aeruginosa* Infections in Cystic Fibrosis

**DOI:** 10.3390/idr17040080

**Published:** 2025-07-07

**Authors:** Camelia Corina Pescaru, Alexandru Florian Crișan, Adelina Marițescu, Vlad Cărunta, Monica Marc, Ștefan Dumitrache-Rujinski, Sorina Laitin, Cristian Oancea

**Affiliations:** 1 Center for Research and Innovation in Personalized Medicine of Respiratory Diseases (CRIPMRD), “Victor Babes” University of Medicine and Pharmacy Timisoara, Eftimie Murgu Square 2, 300041 Timisoara, Romania; pescaru.camelia@umft.ro (C.C.P.); marc.monica@umft.ro (M.M.); oancea@umft.ro (C.O.); 2 Pulmonary Rehabilitation Center, Clinical Hospital of Infectious Diseases and Pulmonology, “Victor Babes”, Gheorghe Adam Street 13, 300310 Timisoara, Romania; adelina.maritescu@umft.ro (A.M.); carunta.vlad@gmail.com (V.C.); 3 Research Center for Assessment of Human Motion, Functionality and Disability, “Victor Babes” University of Medicine and Pharmacy Timisoara, Eftimie Murgu Square 2, 300041 Timisoara, Romania; 4 Doctoral School, “Victor Babes” University of Medicine and Pharmacy Timisoara, Eftimie Murgu Square 2, 300041 Timisoara, Romania; 5 Pulmonology Clinic, Clinical Hospital of Infectious Diseases and Pulmonology, “Victor Babes”, Gheorghe Adam Street 13, 300310 Timisoara, Romania; 6 Pulmonology Department, University of Medicine and Pharmacy “Carol Davila”, Dionisie Lupu nr.37, Sector 1 Bucharest, 030167 Bucharest, Romania; stefan.dumitrache@umfcd.ro; 7 Epidemiology University Clinic, Department XIII, “Victor Babes” University of Medicine and Pharmacy Timisoara, Eftimie Murgu Square 2, 300041 Timisoara, Romania; laitin.sorina@umft.ro; 8 Epidemiology Department, Clinical Hospital of Infectious Diseases and Pulmonology, “Victor Babes”, Gheorghe Adam Street 13, 300310 Timisoara, Romania

**Keywords:** *Pseudomonas aeruginosa*, cystic fibrosis, modulator therapy, biofilms, lung infections

## Abstract

**Background**: Cystic fibrosis (CF) is an autosomal recessive disease caused by mutations in the cystic fibrosis transmembrane conductance regulator (CFTR) gene. Modulator therapies have the ability to improve CFTR function in CF patients, but despite the clear evidence of benefits regarding CFTR modulator therapy, including improved lung function, the reduced rate of exacerbations, and an overall improved quality of life, studies focusing on the reduction rates of *P. aeruginosa* infections during modulator therapy expressed the need for future research on this topic. **Objective**: This study aimed to evaluate the impact of CFTR modulator therapies on the prevalence, density, and persistence of *P. aeruginosa* infection in CF patients and to explore the mechanisms involved. **Methods**: A systematic literature review was performed by searching five major databases (PubMed, Cochrane Library, Scopus, Google Scholar, and Web of Science), and 21 relevant articles investigating the link between CFTR therapy and *P. aeruginosa* infections were selected following the PRISMA guidelines. **Results**: The data indicated that Ivacaftor and the combination Elexacaftor/Tezacaftor/Ivacaftor (ETI) can reduce total bacterial load and markers of systemic inflammation. However, clonal lines of *P. aeruginosa* persist in most cases, and complete eradication is rare, mainly due to biofilm formation and antimicrobial resistance. **Conclusions**: Although CFTR-modulating therapies help to improve clinical condition and reduce inflammation, they do not consistently lead to the elimination of *P. aeruginosa*.

## 1. Introduction

Cystic fibrosis (CF) is an autosomal recessive disease caused by mutations in the cystic fibrosis transmembrane conductance regulator (CFTR) gene, expressed on the apical surface of epithelial cells, which regulates ion transport through chloride channels [1]. The dysfunction of the CFTR protein leads to ion imbalance, the depletion of airway surface fluid, and pH alteration, thus impairing mucociliary clearance and host immune defenses, ultimately leading to higher susceptibility to chronic airway infections. CFTR dysfunction induces thick mucus accumulation in the airway lumen, alteration in airway microenvironment with airway surface dehydration, and impaired mucociliary clearance [2,3].

A major opportunistic pathogen that causes recurring pulmonary infections in patients diagnosed with CF is *Pseudomonas aeruginosa*. Its infections are associated with a higher morbidity and mortality rate in many demographic groups, including in patients with pneumonia, chronic obstructive pulmonary disease (COPD), and CF [4]. The World Health Organization (WHO) included it on its priority list of bacterial pathogens, for which the research and development of new strategies are needed [5]. *P. aeruginosa* is highly capable of causing both acute and chronic infections. Its pathogenic profile comes from a variable and broad depository of virulence factors and antibiotic resistance stored in its genome. Infections with this pathogen are correlated with increased pulmonary exacerbations, accelerated decline in lung function, impaired quality of life, and early death in patients with CF [4,6].

Under normal physiological conditions, CFTR operates as a cAMP-regulated chloride and bicarbonate channel and is upregulated at the apical plasma membrane of various epithelial tissues. It has a key role in maintaining electrolyte and fluid balance, therefore regulating the composition and volume of epithelial secretions [7]. In a mutation in the CFTR gene, CFTR protein expression and/or function is impaired, leading to abnormal ion transport and dehydration of the epithelial surface [7]. This process leads to thick mucus accumulation, chronic inflammation, and recurrent infection, which over time causes tissue damage and structural remodeling [7,8].

Modulator therapies have the ability to improve CFTR function and mutant CFTR protein in CF patients and are classified into the following five main categories according to their respective effects on CFTR mutations: enhancers, correctors, stabilizers, read-through agents, and enhancers [9].

Recent CFTR modulators have revolutionized CF care, correcting defects, improving health, and slowing disease progression. Highly effective CFTR modulator therapy, including Elexacaftor/Tezacaftor/Ivacaftor (ETI), has been found to reduce significantly upper and lower respiratory symptoms and is approved for up to 90% of adults with CF genetic disorders [10,11,12].

Despite the clear evidence of benefits regarding CFTR modulator therapy, including improvement in lung function, a reduced rate of exacerbations, and an overall improved quality of life, observational studies focusing on the interaction between *P. aeruginosa* and CFTR modulators demonstrate uncertain results [13,14].

This study aimed to investigate the infection rates of *P. aeruginosa* during CFTR modulator therapy in patients with cystic fibrosis. By conducting a systematic review, we wanted to assess whether CFTR modulators influence the frequency of respiratory infections caused by *P. aeruginosa*, which is known to be difficult to treat.

## 2. Materials and Method

The narrative systematic review follows the PRISMA guidelines (Preferred Reporting Items for Systematic Reviews and Meta-Analyses), which ensures methodological transparency, reproducibility, and comprehensive reporting of the search strategy, selection process, data extraction, and findings synthesis.

We conducted a multi-step process to analyze the literature regarding *P. aeruginosa* infections during CFTR modulator therapy.

### 2.1. Search Strategy

Our study included a comprehensive current literature search conducted across multiple online databases (PubMed, Cochrane Library, Scopus, Google Scholar, Web of Science) in order to identify potentially relevant studies related to our topic of interest. The search strategy also employed medical subject headings, such as “*P. aeruginosa*”, “CFTR modulators”, “biofilms”, and “cystic fibrosis”.

### 2.2. Eligibility Criteria

**Figure 1:**
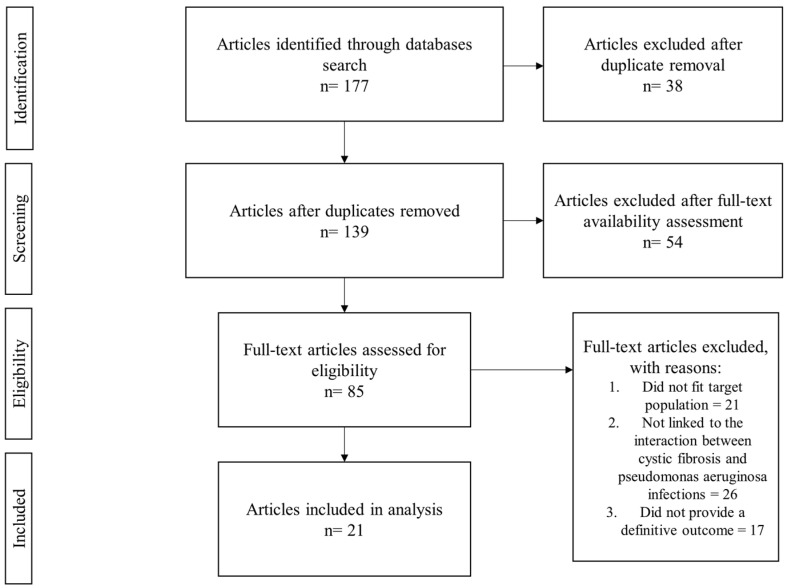
Flowchart of the selection process.

The inclusion criteria were predefined to include studies that focused on the interaction between CFTR modulator therapy and *P. aeruginosa* infections in CF patients and were assessed based on this criteria during our initial screening. Once the search results were retrieved, we excluded duplicates, studies older than 10 years, and those that did not provide full-text availability. Titles and abstracts were screened for relevance. Studies that did not meet our inclusion criteria at this stage were excluded. Full-text articles of eligible studies were later retrieved and assessed, while focusing on their study design, population, intervention process, and outcomes (as expressed in Figure 1). Our selection process aimed at ensuring that the systematic review provided a comprehensive and unbiased analysis of available literature.

## 3. Results

### 3.1. Article Characteristics

At the end of our selection process, 21 studies were included in this review. Out of the 21 articles, 6 were observational cohort studies (28.57%) [15,16,17,18,19,20], 5 were randomized controlled trials (23.80%) [10,13,14,21,22], 3 were longitudinal cohort studies (14.28%) [23,24,25], 2 were prospective monocentric studies (9.52%) [11,26], prospective observational studies (9.52%) [12,27], and experimental studies (9.52%) [28,29], and 1 was a qualitative analysis study (4.76%) [30].

A diverse range of treatment modalities for culture-positive *P. aeruginosa* infections and CFTR modulator therapy were assessed, as described in Table 1.

### 3.2. Protocol

During our assessment of the studies, we identified and outlined the protocols used in the selected articles. The most frequent protocol used was Elexacaftor/Tezacaftor/Ivacaftor (46.61% [10,11,12,13,14,15,16,21,27], followed by Ivacaftor (9.52%) [17,23], with the following protocols: ex vivo analyses of Ki-67 expression in antigen-specific CD154 (+) T cells [25], Ivacaftor, and antibiotics [18]; rRNA gene amplicon sequencing of sinus, throat, and sputum samples [19]; the physiological effects of ETI [30]; antibiotic treatment to combat *pseudomonas* biofilms [22]; Ivacaftor, Ivacaftor/Lumacaftor, Tezacaftor/Ivacaftor [24]; Symkevi/ETI [26]; Ivacaftor, Lumacaftor, Tezacaftor, Elexacaftor, and ETI combined with antibiotics [28]; phage and ciprofloxacin alone and in combination [29]; and Orkambi [20] being tied at 4.76%.

Assessment of methodological quality and reliability of evidence.

The quality of the selected studies was assessed using the JBI (Joanna Briggs Institute) tool, adapted according to each type of study. Each tool contains between 8 and 13 items, covering internal validity, the selection of participants, the measurement of exposure and outcome, and the management of confounding factors. A score was calculated for each study and was expressed as the ratio between the number of items that met the criteria and the total number of applicable items. The JBI scale has a system of scores that consist of ≥8/10 or ≥10/13, which indicate good quality, 7/10, which expresses moderate quality, and scores ≤ 6/10, which suggest a high risk of bias [31].

The GRADE (Grading of Recommendations, Assessment, Development and Evaluation) scale was used to assess the level of confidence in the included studies. This grading classifies the overall certainty of evidence into the following four main categories: high, moderate, low, and very low, which take the risk of bias, inconsistency of results, indirectness of evidence, imprecision of estimates, and risk of selective publication into account. The GRADE scale has a grading that consists of a high score, which indicates a strong evidence that the study was not affected by significant limitations, while a low or very low score reflects uncertainty about the estimated effect [32].

The evaluation was conducted by two separate authors, and discrepancies were discussed until a consensus was reached.

### 3.3. Study Group Demographics

Out of the 21 selected studies that employed a study group, there were a total of 4332 patients with CF registered, with a distribution of 48% female and 49% male, with the remaining 3% being the number of patients with unspecified sex. We found that among these patients, 3008 had at least one copy of the F508del CFTR mutation, making up for 69.4% of the total registered patients. This result is in line with other studies regarding the frequency of the F508del, it being the most common mutation [33,34,35].

### 3.4. Description of Studies

#### 3.4.1. ETI

In a study conducted by Lee T. et al. that involved 468 patients, regarding the annual rate of lung function decline of CF patients on ETI treatment, discovered that on average, pulmonary function was not lost over a two-year period, assessed through a mean annualized rate of change in percent predicted forced expiratory volume in 1 s (ppFEV_1_), thus demonstrating that CFTR modulator therapy has the potential of stopping lung function decline over an extended period of time in CF patients [15].

Ledger E. et al. conducted a randomized controlled trial, involving 11 patients with CF, to investigate how *P. aeruginosa* in CF patients may change in an altered lung environment after the initiation of CFTR therapy. They showed that clonal lineages of *P. aeruginosa* persisted even after CFTR therapy, with no evidence of displacement by alternative strains, sustained mucoid morphology, and continued resistance to antibiotics in isolates [21].

Long D. et al. investigated through a randomized controlled trial involving 15 patients with CF, of which 10 were culture-positive with *P. aeruginosa*, whether methods for this pathogen whole genome hybridization enrichment could enhance detection from cfDNA. Read counts of *P. aeruginosa* for the 10 culture-positive patients increased by 3505-fold on average, thus indicating that the sequencing power can potentially be reduced by that same factor without a negative impact on assay performance, but relative levels of normalized *P. aeruginosa* cfDNA remained unchanged when compared among patients. Their results express that plasma cfDNA sequencing can identify *P. aeruginosa* respiratory culture positivity in CF patients, even those treated with CFTR modulators [10].

In a prospective monocentric study conducted by Schnell A. et al., 69 patients were evaluated on the effects of ETI treatment on clinical, biochemical data, and *P. aeruginosa* colonization rate. Marked improvements on biochemical markers of systemic inflammation were observed, as white blood cell count and the level of immunoglobulin A, G, M, and albumin within 24 weeks of therapy were monitored. The authors concluded that ETI treatment was effective in ameliorating lung function and sweat chloride concentration. Colonization status of *P. aeruginosa* assessment revealed a conversion from a positive to negative detection in 36% of cases after a one-year period of therapy [11].

Similar results were obtained in a prospective observational study ran by Migliorisi G. et al., who enrolled 13 patients with CF, with the aim of defining the clinical and microbiological implications of ETI treatment administration. They reported that airway infection rates decreased, and pulmonary exacerbations were drastically reduced after a one-year period of therapy; however, *P. aeruginosa* showed continuous colonization rates, although slightly reduced [27].

One study conducted by Aspinall S. et al. discusses the lived experience of 12 CF patients undergoing ETI therapy and the psychological aspects involved to determine the disease burden during CFTR therapy. They concluded that individuals undergoing ETI therapy experience increased anxiety and fear of returning to life pre-ETI treatment [30].

Sutharsan S. et al. employed an observational cohort study that aimed at evaluating the real-world impact of ETI on lung function, pulmonary exacerbations frequency, sweat chloride concentration, and nutritional status on 2645 CF patients. Over the first year of ETI, they observed an increase in ppFEV_1_ by 11.3%, a decrease of 75.9% in pulmonary exacerbation frequency, and a decrease in mean sweat chloride concentration of 50.9 mmol/L [16].

#### 3.4.2. Ivacaftor

Rowe S. et al. conducted a longitudinal cohort study that involved 151 patients with CF and expressed significant clinical and physiologic improvements on the initiation of Ivacaftor, with an improvement in predicted FEV_1_% (forced expiratory volume in 1 s) from baseline to 6 months, an improvement in baseline body mass index (BMI), decreased sweat chloride from baseline to 6 months, and a reduction in *P. aeruginosa* infection [23].

In an observational cohort study by Durfey S. et al., involving 10 patients with CF, the combination of Ivacaftor and an intensive three and a half months of antibiotic course was investigated on the impact on chronic *P. aeruginosa* clearance. Ivacaftor alone improved CFTR activity and lung function and inflammation within 48 h and reduced *P. aeruginosa* density by ~10 fold within a week. However, at the end of the study, they concluded that all persistently *P. aeruginosa* culture-positive CF patients remained infected by their pretreatment strain, suggesting that chronic CF infection with this pathogen resist eradication even after marked and rapid modulator-induced improvements in lung infection and inflammation parameters and aggressive antibiotic treatment [18].

Heltshe S. et al. enrolled 151 CF patients in a longitudinal observational cohort study to examine changes in CF respiratory pathogens with Ivacaftor and the correlation with baseline characteristics and their clinical response. Of the 89 patients that were culture-positive for *P. aeruginosa* the year prior to Ivacaftor use, 26 were culture-negative the year following treatment, with 52 other culture-negative patients remaining uninfected. They showed a 35% reduction in *P. aeruginosa* positivity in the year after Ivacaftor treatment, compared to the year prior, also showing reduced odds of mucoid *P. aeruginosa* and *Aspergillus* but not *S. aureus* or other common CF pathogens [17].

Westholter D. et al. conducted a longitudinal cohort study with peripheral blood mononuclear cells and serum samples collected from 108 patients with CF in order to evaluate if CFTR modulator therapy also targets T cells and thereby influences immune cell abnormalities in CF. They concluded that *P. aeruginosa* impairs regulatory T cells in CF patients [24].

#### 3.4.3. Experimental

Eschenhagen P. et al. performed ex vivo analyses of Ki-67 expression in antigen-specific CD154 (+) T cells against bacterial and fungal respiratory pathogens in CF after the initiation of highly effective CFTR modulator therapy and showed a significant decrease in mean Ki-67 expression in antigen-specific CD154 (+) T cells against *P. aeruginosa*, *Aspergillus fumigatus*, *Scedosporium apiospermum*, and *Candida albicans*, but not *Staphylococcus aureus* or mean total serum IgG and IgE, and they showed a significant increase in BMI and FEV_1_ after the initiation of ETI treatment [25].

The same results were registered in another observational cohort study conducted by Armbruster C. et al. with a cohort of 19 CF patients. They expressed that patients remained infected throughout their upper and lower respiratory tract with the same strain of *P. aeruginosa* after the initiation of ETI treatment, and that those strains continued to evolve in response to the newly CFTR-corrected airway [19].

#### 3.4.4. Orkambi

In an observational cohort study by Adam D. et al., 22 CF patients were observed and evaluated during Orkambi combination treatment for the effects on the repair of the CF primary airway epithelia in infectious conditions. Their results showed that combined treatment with VX-809 and VX-770 contributed to a greater beneficial impact on airway epithelial repair and a slight improvement in airway epithelial repair and transepithelial resistance, even in the presence of *P. aeruginosa* exoproducts [20].

#### 3.4.5. Biofilm

A randomized controlled trial by Yau Y. et al. that enrolled 88 patients with CF aimed to determine whether *P. aeruginosa*’s antimicrobial susceptibility testing grown as a biofilm, instead of planktonically, improves the efficacy of antibiotic treatment on pulmonary exacerbations. Their results show that biofilm antimicrobial susceptibility testing did not improve microbiological or clinical outcomes compared to the conventional methods of treatment of pulmonary exacerbations in CF patients with chronic *P. aeruginosa* infection [22].

## 4. Discussion

Impact of CFTR modulators on *P. aeruginosa* colonization.

Our study aimed to investigate the infection rates of *P. aeruginosa* during CFTR modulator therapy in patients with cystic fibrosis. *P. aeruginosa* displays a resistance to a wide variety of antibiotics. Typically, *P. aeruginosa* has three primary mechanisms used to suppress antibiotics, which can be classified as intrinsic resistance, acquired resistance, and adapted resistance [36]. Intrinsic resistance refers to its low-outer membrane permeability and through antibiotic expulsion out of the cell using efflux pumps, leading to enzymes that inactivate antibiotics [4,37]. Acquired resistance is expressed through multifactorial mutational change or chromosomal mutation and the capability of the horizontal transfer of resistance genes [38,39]. Lastly, its adaptive resistance leads to the formation of sputum-suspended aggregates, also named biofilms, in the patient’s lungs, where it serves as a barrier, thus limiting antibiotic access to bacterial cells [36,40]. Current literature reports a 36% conversion from a positive *P. aeruginosa* to a negative *P. aeruginosa* status following 12 months of ETI treatment and also a 35% reduction in *P. aeruginosa* mucoid detection following Ivacaftor therapy [11,17]. However, *P. aeruginosa* long-term persistence remains high [29,37,39,41].

Persistence of colonies and biofilm as a therapeutic barrier.

Sputum-suspended aggregates, or biofilms, consist of matrix-associated exopolysaccharides (EPS), extracellular DNA, and proteins [40,41]. This structure produces chemical and nutrient gradients that affect cells differently. It also provides physical protection against antimicrobials and immune host cells [42]. Biofilm-grown *P. aeruginosa* has a constant and gradual adaptation that bolsters its defensive capabilities and survivability. In cases where a failed attempt at its eradication occurs, the infection becomes chronic and leads to further inflammation and scarring [42]. This often leads to a decrease in lung function, quality of life, and increased mortality in infected cystic fibrosis patients [38,43]. After the initial colonization of the airways, *P. aeruginosa* transitions from a planktonic to a biofilm-like phenotype, particularly under the influence of a microenvironment with low pH, hypoxia, and osmotic stress [44]. This transition is associated with a profound bacterial transcriptomic reprogramming, including the activation of the las and rhl regulatory systems, as well as the overexpression of the algD gene, responsible for alginate synthesis. Post-transplant histopathologic studies have shown that areas of increased biofilm density correspond topographically with regions of severe bronchiectasis and parietal pulmonary fibrosis, supporting the idea that biofilm is not only an effect of chronic infection but an active factor in tissue progression [45].

This chronic picture perpetuates a vicious circle; the biofilm maintains the inflammation, which in turn, sustains the survival of the biofilm. Therefore, therapeutic strategies combining CFTR modulators with anti-biofilm agents (such as DNase, quorum sensing inhibitors or bacteriophages) are promising but still under-tested in randomized clinical trials [46].

Current research gaps and limitations of included studies.

Until 2012, CF therapies were mainly focused on disease sign and symptom management through inhalation and physical therapy alongside numerous daily medications, including antibiotics, anti-inflammatory agents, and mucolytics, assessed by a multidisciplinary healthcare team [47,48,49]. With the help of fundamental advances in the development of preclinical cell models and the implementation of cell-based high-throughput screening essays, new treatment modalities appeared that target the primary CFTR defect [50]. These CFTR modulators can restore both the folding and cross-linking of the mutant CFTR protein or increase the probability of channel opening when the protein is localized to the plasma membrane [20,21,25].

As expressed in our study assessment process, we outlined the most frequently used CFTR modulator protocols, the main ones being the triple combination of Elexacaftor–Tezacaftor–Ivacaftor, followed by Ivacaftor-only treatment. Due to the relatively recent introduction of CFTR modulators, there is currently a lack of prospective observational and experimental studies that assess the interaction between these modulators and *P. aeruginosa*.

Ivacaftor was the first CFTR potentiator that expressed clinically significant improvements in lung function and nutritional status in patients with cystic fibrosis [17,23]. It was approved by the FDA for numerous CFTR residual mutations in in vitro studies [51], with its clinical benefits confirmed in several clinical studies [17,52,53]. However, a couple of studies reported that indeed Ivacaftor treatment showed significant clinical and physiological improvements in CF patients, but not only do these patients remain colonized with *P. aeruginosa*, they also show that the same clonal lineages persist, as opposed to eradicating the preexisting strains [21,23]. Of note, despite reporting an improved airway obstruction, the biomarkers of airway inflammation showed no meaningful improvement with the addition of Ivacaftor [25]. One likely explanation of this infection persistence is the irreversible structural damage that causes defective pathogen clearance, with *P. aeruginosa* being known for its adaptability to a CF lung environment [27].

The triple combination, or ETI, is the first triple combination of modulator drugs approved for cystic fibrosis patients aged two or higher with at least one F508del mutation. This mutation is the most common among patients with CF, making it accessible for most patients [54]. There continues to be a gap in our understanding of whether or how these CFTR modulators affect the microbiological profile in CF patients.

While Ivacaftor showed an initial reduction in the sputum and isolation of bacterial pathogens, Heltshe et al. showed that following Ivacaftor treatment, *P. aeruginosa* detection rates were decreased by 35% over the course of one year, with 26 out of 89 culture-positive patients becoming culture-negative [17]. Similar results were expressed by Durfey et al., who observed a ten-fold reduction in *P. aeruginosa* density following one week of Ivacaftor treatment; however, long-term eradication was not achieved [18]. *P. aeruginosa* density and strains rebounded and were still present even after intensive antibiotic therapy [17,18,54]. In similar studies examining ETI’s impact on *P. aeruginosa* infections, several researchers showed a decrease in total bacterial load and a normalization of systemic inflammation markers, reflected by a reduction in *P. aeruginosa* RNA quantity in the sputum samples of CF patients following ETI treatment [11,19,28]. However, as in the case of Ivacaftor, although a reduction in bacterial load was recorded, some studies found that 100% of involved patients remained colonized with the same strain of *P. aeruginosa* even after six to twelve months of CFTR therapy [19,21]. Additional research is needed to determine how CF patients continue to change post-ETI treatment.

Therapeutic implications and combined strategies.

Molecular synergy between inhaled antibiotics and CFTR modulators has been suggested in the literature [18,55]. However, current evidence does not fully support this hypothesis. One study that evaluated the bacterial density of *P. aeruginosa* reported a decrease in CF patients receiving CFTR modulators with concurrent inhaled antibiotics, with very few patients reporting a cleared infection [18]. Currently, there is insufficient data to support the discontinuation or continuation of inhaled antibiotic therapy during CFTR modulator therapy. Additional data is needed to develop clear guidelines.

Translational perspectives and future research directions.

New treatments are emerging with the hope of improving the CFTR function, pulmonary function, and overall quality of life of CF patients. Recent studies involving a new triple therapy of Vanzacaftor–Tezacaftor–Deutivacaftor show promising results in this field [56,57,58].

Vanzacaftor–Tezacaftor–Deutivacaftor therapy has been approved for CF patients aged six and above, demonstrating significant increases in lung function and the correction of the defective CFTR protein, alongside a reduction in sweat chloride levels [59].

Two phase-three studies demonstrated non-inferiority to ETI with significant improvements in both lung function, through a substantial increase in ppFEV_1_ in the studied population, and the correction of CFTR proteins [60,61]. Whereas Ivacaftor treatment is taken twice a day, the combination of Vanzacaftor–Tezacaftor–Deutivacaftor is recommended once a day, potentially improving the adherence to prescribed treatment in CF patients [62,63]. However, infection rates in the target population were not studied; thus, further research into this topic is needed to determine if this new therapy also reduces infection rates caused by *P. aeruginosa* in CF patients.

## 5. Conclusions

*P. aeruginosa* infections remain a recurrent problem in CF, even in the context of next-generation CFTR modulator therapies. Although reviewed studies show a reduction in bacterial load and improvement in inflammatory markers following treatment with Ivacaftor or ETI, the complete eradication of the bacteria is rarely achieved, and persistent colonies continue to affect patients.

These findings highlight the need for further research into the connection between CFTR modulators and the resistance mechanisms of *P. aeruginosa*, analyzing both the role of biofilm and chronic inflammation in maintaining colonization rates.

Integrating both anti-biofilm and modulator therapy into a personalized therapeutic program could lead to an improvement in the control of chronic infections and a higher quality of life.

## Figures and Tables

**Table 1:** Article protocol and outcome characteristics.

Authors	Design	Protocol	Outcomes	JBI	GRADE
**Lee et al.** [15]	Observational cohort study	Elexacaftor/Tezacaftor/Ivacaftor (ETI)	Colonization: not reported; Lung function: stable 2 years; Markers: not reported.	7/10	Moderate
**Ledger et al.** [21]	Randomized controlled trial	Elexacaftor/Tezacaftor/Ivacaftor (ETI)	Colonization: persistent; Resistance: unchanged; Biofilm: maintained after CFTR modulator therapy.	9/10	Low
**Long et al.** [10]	Randomized controlled trial	Elexacaftor/Tezacaftor/Ivacaftor (ETI)	Colonization: detectable by cfDNA; Relative cfDNA level: unchanged.	8/10	Moderate
**Schnell et al.** [11]	Prospective monocentric study	Elexacaftor/Tezacaftor/Ivacaftor (ETI)	Colonization: 36% negative conversion; Lung function: improved; Markers: ↓leukocytes, Ig.	9/10	Moderate
**Migliorisi et al.** [27]	Prospective observational study	Elexacaftor/Tezacaftor/Ivacaftor (ETI)	Colonization: slight↓; Lung function: improved; Exacerbations: decreased.	8/10	Moderate
**Aspinall et al.** [30]	Qualitative analysis study	Physiological effects of ETI	Colonization: not reported; Subjective effects: ↑anxiety, improved quality of life.	8/10	Low
**Sutharsan et al.** [16]	Observational cohort study	Elexacaftor/Tezacaftor/Ivacaftor (ETI)	↓Colonization; Lung function: ↑FEV1; Exacerbations: ↓75.9%; Markers: ↓sweat chloride.	9/10	High
**Middleton et al.** [13]	Randomized controlled trial	Elexacaftor/Tezacaftor/Ivacaftor (ETI)	↓Colonization; Lung function: ↑FEV1; Markers: ↑CFQ-R, ↓sweat chloride.	9/10	High
**Heijerman et al.** [14]	Randomized controlled trial	Elexacaftor/Tezacaftor/Ivacaftor (ETI)	↓Colonization; Lung function: ↑FEV1; Markers: ↓sweat chloride.	9/10	High
**Nichols et al.** [12]	Prospective observational study	Elexacaftor/Tezacaftor/Ivacaftor (ETI)	↓Colonization; Lung function: ↑FEV1, BMI; Exacerbations: ↓.	8/10	High
**Rowe et al.** [23]	Longitudinal cohort study	Ivacaftor	↓Colonization; Lung function: ↑FEV1; Clinical markers: ↑BMI, ↓hospitalizations.	8/10	Moderate
**Heltshe et al.** [17]	Observational cohort study	Ivacaftor	Colonization: ↓35% mucoid; Lung function: improved;	8/10	Moderate
**Durfey et al.** [18]	Observational cohort study	Ivacaftor and antibiotics	Colonization: ↓density; Eradication: Still present; Lung function: ↑FEV1.	8/10	Moderate
**Westholter et al.** [24]	Longitudinal cohort study	Ivacaftor; Ivacaftor/Lumacaftor; Tezacaftor/Ivacaftor	↓Colonization; T regulatory cells influenced; Lung function: partially improved.	9/10	Moderate
**Cigana et al.** [28]	Experimental study	Ivacaftor, Lumacaftor, Tezacaftor, Elexacaftor, and ETI combined with antibiotics on sequential CF isolates	Colonization: ↓density; Antibiotics + modulators: partial synergy.	8/10	Moderate
**Armbruster et al.** [19]	Observational cohort study	rRNA gene amplicon sequencing of sinus, throat, and sputum samples before and after initiation of ETI	Colonization: persistent; Same strain in upper and lower tracts.	7/10	Low
**Eschenhagen et al.** [25]	Longitudinal cohort study	Ex vivo analyses of Ki-67 expression in antigen-specific CD154 (+) T cells against *P. aeruginosa*	Colonization: not reported; Immunological markers: ↓activation of B and T lymphocytes.	8/10	Moderate
**Yau et al.** [2]	Randomized controlled trial	Antibiotic treatment to combat *P. aeruginosa* biofilms	Colonization: persistent; Biofilm-guided antibiotics: limited efficacy.	8/10	Low
**Ahmed et al.** [26]	Prospective monocentric study	Symkevi/ETI	Colonization: no significant changes; Pulmonary function: not reported.	7/10	Low
**Luscher et al.** [29]	Experimental study	Phage and ciprofloxacin alone and in combination to treat *P. aeruginosa* infections in an ex vivo human airway epithelial cell line model	Colonization: ↓with phage + antibiotic combination; Ex vivo model.	8/10	Low
**Adam et al.** [20]	Observational cohort study	Orkambi	Colonization: slight↓; Lung function: partially improved; ↑Epithelial repair.	9/10	Moderate
Table legend: ↑—increase; ↓—decrease					

## Data Availability

Not applicable.

## Abbreviations

The following abbreviations are used in this manuscript:

CF	Cystic Fibrosis
CFTR	Cystic Fibrosis Transmembrane Conductance Regulator
COPD	Chronic Obstructive Pulmonary Disease
WHO	World Health Organization
ETI	Elexacaftor–Tezacaftor–Ivacaftor
EPS	Exopolysaccharides
FEV_1_	Forced Expiratory Volume in 1 s
GRADE	Grading of Recommendations, Assessment, Development, and Evaluation
JBI	Joanna Briggs Institute
ppFEV_1_	Percent Predicted Forced Expiratory Volume in 1 s
PRISMA	Preferred Reporting Items for Systematic Reviews and Meta-Analyses
